# Surprising similarities in model and observational aerosol radiative forcing estimates

**DOI:** 10.5194/acp-20-613-2020

**Published:** 2020-01-17

**Authors:** Edward Gryspeerdt, Johannes Mülmenstädt, Andrew Gettelman, Florent F. Malavelle, Hugh Morrison, David Neubauer, Daniel G. Partridge, Philip Stier, Toshihiko Takemura, Hailong Wang, Minghuai Wang, Kai Zhang

**Affiliations:** 1 Space and Atmospheric Physics Group, Imperial College London, London, UK; 2 Institute for Meteorology, Universität Leipzig, Leipzig, Germany; 3 National Center for Atmospheric Research, Boulder, USA; 4 College of Engineering Mathematics and Physical Sciences, University of Exeter, Exeter, UK; 5 Met Office, Fitzroy Road, Exeter, UK; 6 Institute for Atmospheric and Climate Science, ETH Zurich, Zurich, Switzerland; 7 Atmospheric, Oceanic and Planetary Physics, Department of Physics, University of Oxford, Oxford, UK; 8 Research Institute for Applied Mathematics, Kyushu University, Fukuoka, Japan; 9 Atmospheric Sciences and Global Change Division, Pacific Northwest National Laboratory, Richland, USA; 10 Institute for Climate and Global Change Research, Nanjing University, Nanjing, China; 11 School of Atmospheric Sciences, Nanjing University, Nanjing, China; 12 Collaborative Innovation Center of Climate Change, Nanjing, China

## Abstract

The radiative forcing from aerosols (particularly through their interaction with clouds) remains one of the most uncertain components of the human forcing of the climate. Observation-based studies have typically found a smaller aerosol effective radiative forcing than in model simulations and were given preferential weighting in the Intergovernmental Panel on Climate Change (IPCC) Fifth Assessment Report (AR5). With their own sources of uncertainty, it is not clear that observation-based estimates are more reliable. Understanding the source of the model and observational differences is thus vital to reduce uncertainty in the impact of aerosols on the climate.

These reported discrepancies arise from the different methods of separating the components of aerosol forcing used in model and observational studies. Applying the observational decomposition to global climate model (GCM) output, the two different lines of evidence are surprisingly similar, with a much better agreement on the magnitude of aerosol impacts on cloud properties. Cloud adjustments remain a significant source of uncertainty, particularly for ice clouds. However, they are consistent with the uncertainty from observation-based methods, with the liquid water path adjustment usually enhancing the Twomey effect by less than 50%. Depending on different sets of assumptions, this work suggests that model and observation-based estimates could be more equally weighted in future synthesis studies.

## 1 Introduction

Acting as cloud condensation nuclei (CCN) and ice nucleating particles (INPs), aerosols can modify the cloud droplet number concentration (*N*_d_) and the ice crystal number concentration (*N*_i_). An increase in *N*_d_ can impact the reflectivity of a cloud (Twomey, 1974), resulting in a cooling effect on the climate known as the radiative forcing from aerosol-cloud interactions (RFaci) or the “Twomey effect”. A change in *N*_d_ may also produce cloud adjustments (Albrecht, 1989; Ackerman et al., 2004), resulting in changes to the cloud fraction (*f*_c_) and the liquid water path (L). Similarly, an aerosol-induced change in *N*_i_ may change ice-cloud properties. The combination of these adjustments and the RFaci is known as the effective radiative forcing from aerosol-cloud interactions (ERFaci). The sign and magnitude of the forcing from cloud adjustments are highly uncertain (Han et al., 2002; Seifert et al., 2015; Gryspeerdt et al., 2016; Malavelle et al., 2017; McCoy et al., 2018), and this uncertainty is a leading contributor to uncertainty in the overall effective radiative forcing from aerosols (ERFaer).

Most global climate models (GCMs) include some form of parameterisation of aerosol-cloud interactions, allowing the ERFaer to be calculated (e.g. Quaas et al., 2009; Ghan et al., 2016). However, uncertainties in the parameterisation of cloud and aerosol processes have led to a large variation in these GCM-based estimates. Satellite and in situ observations can be used to constrain the magnitude of the ERFaci, typically focusing on the sensitivity of cloud properties to aerosol perturbations (e.g. Feingold, 2003; Kaufman et al., 2005; Quaas et al., 2008; Gryspeerdt et al., 2017; McCoy et al., 2017). These sensitivities can be either used directly to calculate components of the ERFaer, such as the RFaci (Quaas et al., 2008), or used to constrain processes in global models, improving estimates of the ERFaer (e.g. Quaas et al., 2006). However, in many cases, uncertainties and biases in observations can lead to systematic errors in these observation-based estimates of aerosol-cloud interactions (e.g. Quaas et al., 2010; Gryspeerdt et al., 2016; Stier, 2016; Schutgens et al., 2017; Christensen et al., 2017).

Model-based estimates of the ERFaer tend to be larger (more negative), with Boucher et al. (2013) providing a range of −0.81 to −1.68 Wm^−2^, compared to −0.45 to −0.95 Wm^−2^ for observation-based estimates. Despite their uncertainties, observation-based studies have previously been given a stronger weight in expert assessments of the ERFaer, leading to smaller overall assessments of the ERFaer (Boucher et al., 2013). Understanding this difference between methods is necessary to improve future estimates of the ERFaer. Uncertainty in the magnitude of the ERFaer comes from three main sources:

S1. *Anthropogenic and natural aerosol properties.* Whilst the present-day (PD) CCN and INP burden can be constrained, the composition of the atmosphere of the preindustrial (PI) earth is much more uncertain, creating a significant source of uncertainty in aerosol forcing estimates (Carslaw et al., 2017).
S2. *The sensitivity of N*_*d*_ *and N*_*i*_ *to an aerosol perturbation.* Most climate models include a parameterisation of the impact of aerosol on *N*_d_ through droplet activation and the associated radiative forcing from aerosol-cloud interactions (RFaci or Twomey effect). Variations in the parameterisation of unresolved vertical velocities between models lead to a strong variation in this sensitivity between climate models, despite the similarity of their aerosol activation parameterisations (Gryspeerdt et al., 2017).
S3. *The adjustment of clouds to a change in N*_*d*_ *or N*_*i*_. The magnitude of cloud adjustments (such as changes in *f*_c_, L, or ice water path) are a significant source of uncertainty. The nature of the representation of adjustments varies between models, with some processes (such as those involving ice) being excluded from many models, leading to a large uncertainty in the magnitude and sign of these adjustments (Heyn et al., 2017).

Isolating these different sources of uncertainty is difficult, complicating the use of observations to reduce model biases. Some observation-based studies aim to constrain the entire ERFaer (e.g. Cherian et al., 2014). However, most studies typically estimate components of the ERFaer due to changes in specific cloud properties, such as the RFaci (e.g. Quaas et al., 2008; Gryspeerdt et al., 2017; McCoy et al., 2018), the change in liquid *f*_c_ (*f*_l_) (Gryspeerdt et al., 2016; Christensen et al., 2017), L (Gryspeerdt et al., 2019), or cloud albedo (Lebsock et al., 2008; Christensen et al., 2017) due to the difficulty in isolating specific processes in the atmosphere. In contrast, model studies are able to isolate the radiative forcing due to aerosol impacts on individual processes (e.g. auto-conversion; Gettelman, 2015; or aerosol absorption; Zelinka et al., 2014) but the coupled nature of cloud properties means that the forcing from the RFaci is generally not extracted from the total ERFaer reported (Boucher et al., 2013).

Existing methods of decomposing the top-of-atmosphere radiation changes between a PI and a PD simulation (ERFaer) into components typically require multiple model simulations with different permutations of model processes activated (e.g. Gettelman, 2015) or repeated calls to the radiation parameterisation, requiring significant modification of the model code (e.g. Mülmenstädt et al., 2019). In contrast, the method presented here requires only a single pair of PI and PD simulations with a minimal set of model output (see the Supplement), allowing it to be applied even to existing model ensembles.

This study presents a method, building on Ghan (2013), for decomposing the ERFaer into changes in the surface albedo, the direct effect of aerosols (RFari), and changes in the cloud albedo (Δ*α*_c_) and fraction (Δ*f*_c_). The changes in cloud properties are separated into contributions from liquid and ice clouds (or high and low clouds if cloud phase is not available). Finally, as the primary controls on liquid cloud albedo are L and *N*_d_ (Engström et al., 2015), the changes in liquid cloud albedo is further separated into two terms: one from L changes and a second from *N*_d_ changes (the RFaci), which assume that all other cloud quantities are held constant. This ERFaer decomposition creates a clearer comparison between model and observational estimates of the ERFaer components using minimal computational time and output. The decomposition is shown to compare well to more sophisticated methods and highlights significant agreements between the aerosol forcing estimates by global models and through observation-based methods.

## 2 Methods

### 2.1 Forcing decomposition

To decompose the aerosol forcing into components, two separate model simulations are required: one with PI aerosol emissions and another with PD emissions. The ERFaer is taken as the difference in top-of-atmosphere (TOA) radiation between these two simulations. Cloudy-sky quantities (*x*_c_) are computed from the all-sky (*x*) and clear sky (*x*_clr_) quantities and the cloud fraction (*f*_c_).

(1) $$x_{\text{c}}=\frac{x-x_{\text{clr}}\left(1-f_{\text{c}}\right)}{f_{\text{c}}}$$

The ERFaer is split into longwave (LW) and shortwave (SW) components. The changes in the SW TOA radiation can be attributed to changes in the cloudy-sky albedo, clear-sky albedo (Δ*α*_clr_), and changes in the cloud fraction (Eq. 3). The change in the longwave component (ΔLW) can be similarly decomposed into a cloudy-sky (ΔOLR_c_), clear sky (ΔOLR_clr_), and cloud fraction change. Throughout this work, a Δ signifies PI to PD changes. NoA (no aerosol) indicates an albedo determined in a clean atmosphere (no radiative effect of aerosol; Ghan, 2013). *F*^↓^ is the TOA incoming solar radiation. Note that all of the steps in this decomposition are performed at the grid-box scale.

(2)  ERFaer =ΔSW+ΔLW,

(3) $$\begin{array}{l} \Delta \text{SW}\approx F^{↓}(\left(1-f_{\text{c}}\right)\Delta \alpha_{\text{clr}}^{\text{NoA}}\;\Delta \text{Surf}\\ \begin{array}{l} +\left(1-f_{\text{c}}\right)\Delta \left(\alpha_{\text{clr}}-\alpha_{\text{clr}}^{\text{NoA}}\right)\\ \left(\text{cloudy-sky contribution}\right) \end{array}\text{ SWariclr}\\ \begin{array}{l} +f_{\text{c}}\Delta \left(\alpha_{\text{c}}-\alpha_{\text{c}}^{\text{NoA}}\right)\\ \left(\text{clear-sky contribution}\right) \end{array}\;{\text{SWari}}_{\text{c}}\\ +f_{\text{c}}\Delta \left(\alpha_{\text{c}}^{\text{NoA}}\right)\;\Delta {\text{SW}}_{\text{c}}\\ +\left(\alpha_{\text{c}}-\alpha_{\text{clr}}\right)\Delta f_{\text{c}})\;\Delta {\text{SW}}_{\text{cf}}, \end{array}$$

(4) $$\begin{array}{l} \Delta \text{LW}\approx \left(1-f_{\text{c}}\right)\Delta {\text{OLR}}_{\text{clr}}\text{ LWarics}\\ +f_{\text{c}}\Delta {\text{OLR}}_{\text{c}}\;\Delta {\text{LW}}_{\text{c}}\\ +\left({\text{OLR}}_{\text{c}}-{\text{OLR}}_{\text{clr}}\right)\Delta f_{\text{c}}\;\Delta {\text{LW}}_{\text{cf}}. \end{array}$$

The terms can then be connected to the decomposition of the aerosol forcing in Boucher et al. (2013). The aerosol direct effect or RFari can be approximated as SWari_cs_ + LWari_cs_. This ignores changes in the surface (ΔSurf) and the impact of aerosol above cloud (SWari_cld_), but it provides a comparable value to the RFari estimated using observations (e.g. Quaas et al., 2008). The remaining terms can then be considered the ERFaci (plus cloudy-sky components of the RFari), with terms due to changes in cloud properties (ΔSW_c_) and cloud amount (ΔSW_cf_).

These cloud terms can be further decomposed into changes in liquid and ice cloud (Eqs. 5–7), resulting in forcings from changes in liquid (ΔSW_cl_) and ice-cloud albedo (ΔSW_ci_) as well as the forcings from changes in cloud fraction (ΔSW_cfl_, ΔSW_cfi_). The liquid cloud albedo is determined using only grid boxes with an ice-cloud fraction of less than 2%. A similar criterion is used for the ice-cloud albedo. The forcing from changes in liquid cloud albedo (ΔSW_cl_, the “intrinsic” forcing; Chen et al., 2014) can then be further decomposed into a forcing from changes in L and a change in *N*_d_. Using the strong dependence of cloud albedo on L (Engström et al., 2015), the ERFaci due to L changes can be determined by a linear regression to determine the sensitivity of liquid cloud albedo to L (Eq. 8), combined with a known PI to PD change in L. Similar results are obtained when using ln L instead of L. The forcing due to *N*_d_ changes (the RFaci) is the residual of liquid cloud albedo forcing with the L forcing removed (Eq. 9).

(5) $$f_{\text{c}}\Delta \alpha_{\text{c}}=f_{1}\Delta \alpha_{1}+f_{\text{i}}\Delta \alpha_{\text{i}},$$

(6) $$\Delta {\text{SW}}_{\text{c}}=\Delta {\text{SW}}_{\text{cl}}+\Delta {\text{SW}}_{\text{ci}},$$

(7) $$\Delta {\text{SW}}_{\text{cf}}=\Delta {\text{SW}}_{\text{cfl}}+\Delta {\text{SW}}_{\text{cfi}},$$

(8) $$\Delta \alpha_{1}^{L}={\frac{\text{d}\alpha_{1}}{\text{d}L}|}_{\text{PD}}\Delta L,$$

(9) $$\Delta \alpha_{1}^{N_{\text{d}}}=\Delta \alpha_{1}-\Delta \alpha_{1}^{L}.$$

In many situations, the ice-cloud fraction includes clouds with a low optical depth. This means that in situations where a thin ice cloud overlies a thick low-level liquid cloud, changes in the low-level liquid cloud albedo might be misattributed as changes in the ice-cloud albedo. To avoid this issue, a threshold in-cloud ice water path (IWP) of 8.7 gm^−2^ is required for a grid box to be classed as an ice-cloud grid box. This threshold is approximately equal to the MODIS cloud mask sensitivity of an optical depth of 0.4 (Ackerman et al., 2008), following the relationship from Heymsfield et al. (2003). The shortwave forcing from these optically thin cases is assigned to underlying liquid clouds, assuming that the ratio of the RFaci to the forcing from L adjustments is the same as in the ice-cloud-free regions. The longwave forcing is assumed to originate from the ice clouds, due to the emissivity of these thin clouds. The sensitivity of the decomposition to the IWP sensitivity is investigated in this work.

Changes in overlying ice cloud create a change in the liquid cloud fraction (*f*_l_), but observational estimates of the forcing from liquid cloud adjustments typically assume no change in the ice-cloud fraction *f*_i_ (Gryspeerdt et al., 2016; Christensen et al., 2017). To get a closer agreement between models and observations, the change in liquid cloud fraction (Δ*f*_l_) is adjusted in the model output for changes in the ice-cloud fraction (Δ*f*_i_) following Eq. (10), assuming that the changes in ice-cloud fraction are uncorrelated to the occurrence of liquid cloud.

(10) $$\Delta f_{1}\mapsto \Delta f_{1}+\Delta f_{\text{i}}\frac{f_{1}}{1-f_{\text{i}}}$$

### 2.2 Datasets

The decomposition is applied to pairs of simulations from the AeroCom and CMIP5 intercomparisons. The simulation pairs have prescribed sea surface temperatures and sea ice, differing only in their aerosol emissions. Three-hourly model output from the AeroCom indirect effect experiment simulations (Zhang et al., 2016; Ghan et al., 2016) is used, with 5-year simulations nudged to present-day meteorology for the years 2006–2010. The CMIP5 models make use of the “sstClim” and “sstClimAerosol” simulations, which are 30-year-long free-running simulations with climatological sea surface temperature (SST) fields. Further details on the AeroCom and CMIP5 models can be found in Zhang et al. (2016) and Zelinka et al. (2014), respectively. As a descendent of the HadGEM2-A and HadGEM3-UKCA models, UKESM1-A (Sellar, 2019) has also been included to provide an additional comparison between different versions of the same model (futher details in Mulcahy et al., 2018). It is run in the same configuration at the AeroCom simulations.

To test the accuracy of the decomposition, two additional sets of model simulations were performed using ECHAM6-HAM2.2. The “anthsca” simulations are the same as the base AeroCom setup but with present-day anthropogenic aerosol emissions scaled by a factor given in the simulation name. While both the aerosol distribution and the parameterisations vary between the models used in this work, the “anthsca” simulations demonstrate the impact of changing the aerosol distribution alone. The CND (constant *N*_d_) simulation replaces the *N*_d_ value used in the autoconversion parameterisation with a climatological value, selected to agree with the global mean *N*_d_ in the full two-moment run. This removes any aerosol-dependent cloud adjustments, such that change in liquid cloud albedo is the result of the Twomey effect alone.

## 3 Results

### 3.1 Decomposition comparisons

The total ERFaer in the AeroCom and CMIP5 models varies from −0.36 to −2.30 Wm^−2^ (Table 1), with the majority of models having a stronger SW component that is partially offset by a smaller positive LW forcing. There is a significant variation in the magnitude and even the sign of the components of the forcing calculated using the method from the previous section (Table S2 in the Supplement). However, the residual of the sum of the components of decomposition compared to the total ERFaer calculated is small (typically less than 10%), increasing confidence in the completeness of the decomposition as each term is calculated independently.

The decomposition in this work also compares well to other methods. By removing the aerosol-dependent cloud adjustments using a climatological *N*_d_ (CND), the RFaci is isolated from the adjustments and is found to be within 10% of the value calculated through the decomposition in this work, with the forcing from the cloud adjustments decreasing to close to zero as the adjustments are removed (Table 2). Similarly, the three components of the ERFaci in liquid clouds determined using the sophisticated partial radiative perturbation (PRP) method (Mülmenstädt et al., 2019) match the results of this work to within 15% (Table 2). There is also a close match in the spatial patterns of the forcing from the components between the different methods (Fig. S2). Due to the variability in the cloud field, a higher threshold of 40 gm^−2^ gives very similar forcing values when using daily mean data for the AeroCom models (not shown), although only the 3-hourly AeroCom data are used in this work. The similarity of the results between methods suggest that the method introduced in this work is capable of accurately identifying the individual components of the ERFaer.

The estimate of the RFaci is also found to be insensitive to the value chosen for the IWP threshold used to identify ice clouds (Table 2). Although there is a significant change in the RFaci when a 1 gm^−2^ threshold is introduced, this is likely due to the occurrence of clouds in the model that have little condensed water and hence are not optically active. However, for larger values of the IWP threshold, the variations in the RFaci are within 10% of the value used in this work. Even with a very large threshold value of 100 gm^−2^, the adjustments as a percentage of the RFaci are within 20% of the best estimate, showing that this method is relatively insensitive to the choice of threshold and hence is a suitable method to account for the effect of thin ice clouds.

### 3.2 The RFaci

**Figure 1.**
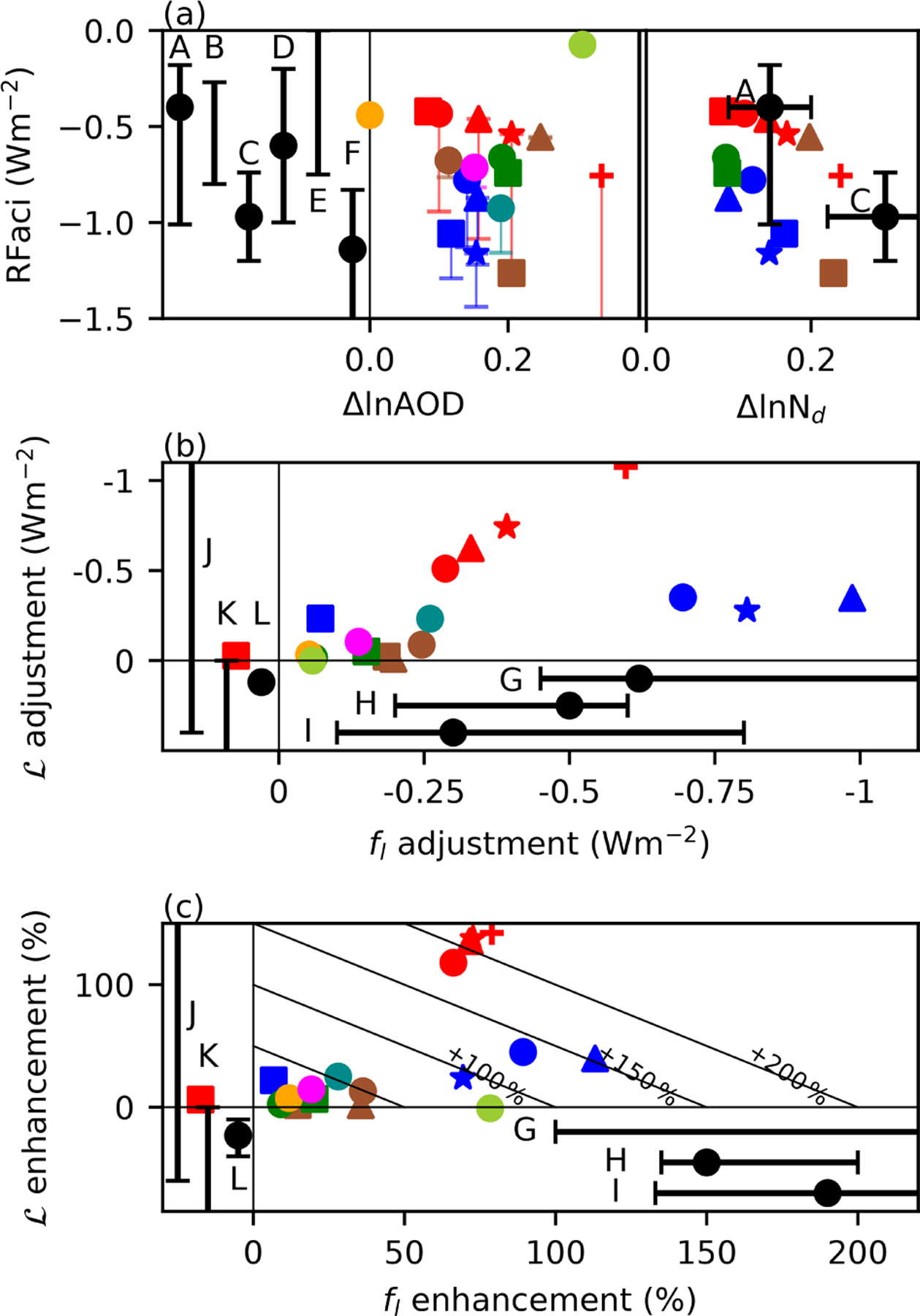
**(a)** The RFaci related to the fractional change in aerosol optical depth (AOD) and *N*_d_. Colours and symbols are given in Table 1; vertical lines link the RFaci estimates to the “intrinsic” (RFaci+LWP adjustment) forcing. The black points are the observation-based estimates from A - Gryspeerdt et al. (2017), B - Fiedler et al. (2017), C - McCoy et al. (2017), D - Bellouin et al. (2013), E - Stevens (2015), and F - Hasekamp et al. (2019). **(b)** Forcing from adjustments in L and liquid *f*_c_. Other estimates from G - Andersen et al. (2017), H - Gryspeerdt et al. (2016), I - Christensen et al. (2017), J - Gryspeerdt et al. (2019), K - Sato et al. (2018), and L - Toll et al. (2019) are shown. Not all studies provide a central estimate (black point). **(c)** The percentage enhancement of the RFaci by L and liquid *f*_c_ changes. Diagonal lines are contours of constant total RFaci enhancement.

Previous observation-based studies estimating the RFaci have used a limited number of methods. A sample of these estimates using various methods and estimates of the anthropogenic aerosol fraction are included in Fig. 1a. A - Gryspeerdt et al. (2017) is representative of studies (e.g. Quaas et al., 2008) using relationships between satellite observations of aerosol and *N*_d_ along with observed cloud properties to convert this to estimate the RFaci. B - Fiedler et al. (2017) use a similar method but incorporate the observed relationship in a climate model to calculate the RFaci (e.g. Quaas et al., 2006). C - McCoy et al. (2017) use reanalysis aerosol instead of observed aerosol properties. D - Bellouin et al. (2013) use a model strongly constrained by satellite observations to estimate the RFaci. E - Stevens (2015) combine several lines of evidence that are distinct from the other studies. F - Hasekamp et al. (2019) use a polarimetric retrieval of aerosol to include more size information and account for the detectability limit in satellite retrievals of aerosol. Although other studies place an implicit limit on the RFaci by constraining the total ERFaer (Cherian et al., 2014) or a combination of the RFaci and the L adjustments (Lebsock et al., 2008; Christensen et al., 2017), they are not included here due to the weak constraint they provide on RFaci. Together the observation-based studies suggest a central estimate for the RFaci in the range −0.2 to −1.0 Wm^−2^ (Fig. 1a).

All the models considered in the present study show a significant ΔSW_c_, typically dominated by changes in liquid clouds (Table S2). This forcing varies significantly, from −0.06 to −1.44 Wm^−2^, outside the range of plausible RFaci generated by many observational constraints (Fig. 1a - crossbars). However, when the forcing due to L adjustments is removed, the variability is reduced, with a lower bound of −1.26 Wm^−2^ and many of the models producing an RFaci estimate around −0.75 Wm^−2^ or smaller (Fig. 1a - markers). Considering the models as a whole, there is a weak relationship between the aerosol optical depth (AOD) perturbation and the RFaci (Fig. 1a), due to the weak relationship between AOD and CCN (Stier, 2016). A stronger relationship between Δ*N*_d_ and the RFaci is seen for the individual models (Fig. 1a), with the remaining variation being due to differences in the cloud field (Zelinka et al., 2014).

**Figure 2.**
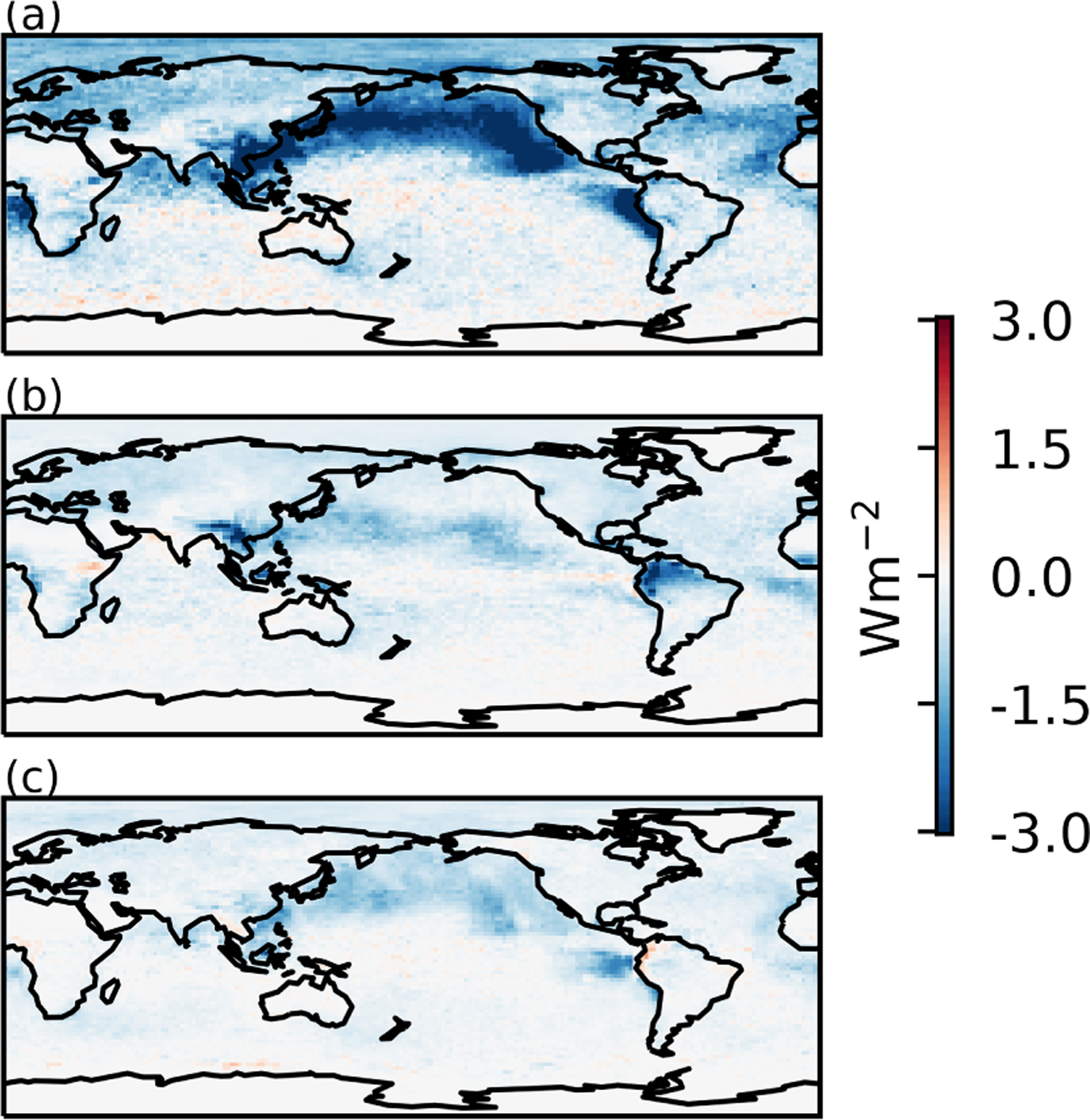
**(a)** The ensemble mean shortwave RFaci. **(b)** ERFaci contribution from *f*_l_ changes. **(c)** ERFaci contribution from L changes.

Global patterns of the RFaci (Fig. 2a) show a weak RFaci over land and stronger effect over the ocean, particularly in regions with large amounts of low cloud. This is very similar to a number of observational estimates, which place the majority of the aerosol forcing over the ocean due to a high *N*_d_ sensitivity to aerosol and *f*_l_ (e.g. Quaas et al., 2008; Gryspeerdt et al., 2017; Christensen et al., 2017).

### 3.3 Liquid cloud adjustments

Uncertainties in the aerosol environment (source S1), droplet activation (S2), and cloud processes (S3) all contribute to the total uncertainty in forcing from liquid cloud adjustments, making model-observation comparisons difficult. However, uncertainties from both S1 and S2 apply to both the RFaci and the adjustments. By reporting cloud adjustments in *f*_l_ and L as a percentage enhancement of the RFaci (Fig. 1c), the impact of S1 and S2 on the estimate of the adjustments can be reduced. This focuses on the uncertainty in the cloud response to *N*_d_ changes (S3), simplifying comparisons between models with different anthropogenic aerosol fractions and activation schemes.

The benefit of normalisation of the adjustments by the RFaci is demonstrated by the analysis of the ECHAM6-HAM ensemble with varying aerosol emissions (ECHAM6-HAM-anthsca, red). Although the forcing from both *f*_l_ and L changes in these simulations is very different (Fig. 1b), the enhancement of the RFaci by both effects is the same to within 10% (Fig. 1c). In contrast, the CAM5 microphysics ensemble (blue) has a similar aerosol environment (Fig. 1a) but very different cloud microphysics schemes for each of its members. As such, the variation in the RFaci enhancement from cloud adjustments is significant among members of this ensemble. This normalisation by RFaci allows the adjustments to be more closely compared with observation-based studies.

#### f_l_ adjustments.

Three recent observational studies using different methods (Gryspeerdt et al., 2016; Andersen et al., 2017; Christensen et al., 2017) find an *f*_l_ adjustment that enhances the RFaci by around 130% to 200%. This remains the case when a different anthropogenic aerosol fraction (MACv2; Kinne, 2019) is used in the Gryspeerdt et al. (2016) estimate. The upper bound to the enhancement in Christensen et al. (2017) is unknown, as the RFaci is not reported separately from L adjustment. This highlights the impact the RFaci uncertainty can have in observational estimates of the enhancement when the RFaci uncertainty is large.

Many of the models, particularly those from CMIP5, have a very small *f*_l_ adjustment, producing an RFaci enhancement close to 0%. This explains the smaller mean forcing from liquid cloud adjustments in Zelinka et al. (2014), where only CMIP5 models were used. The largest model estimates of *f*_l_ adjustments are of a similar magnitude to the observational estimates, with an enhancement of around 100%. While some models are more similar to the observation-based *f*_l_ adjustment forcing (Fig. 1b) than the *f*_l_ enhancement (Fig. 1c), this is due to the model RFaci estimates typically being stronger than the average observation-based estimates (Fig. 1a). The overall pattern of the forcing from *f*_l_ changes in models (Fig. 2b) is similar to that from Gryspeerdt et al. (2016), with a stronger forcing around the edges of the stratocumulus regions, but a weaker forcing in the North Pacific. This is likely related to the mean-state *f*_l_, as increasing the *f*_l_ is difficult if the *f*_l_ is already high.

#### L adjustments.

**Figure 3.**
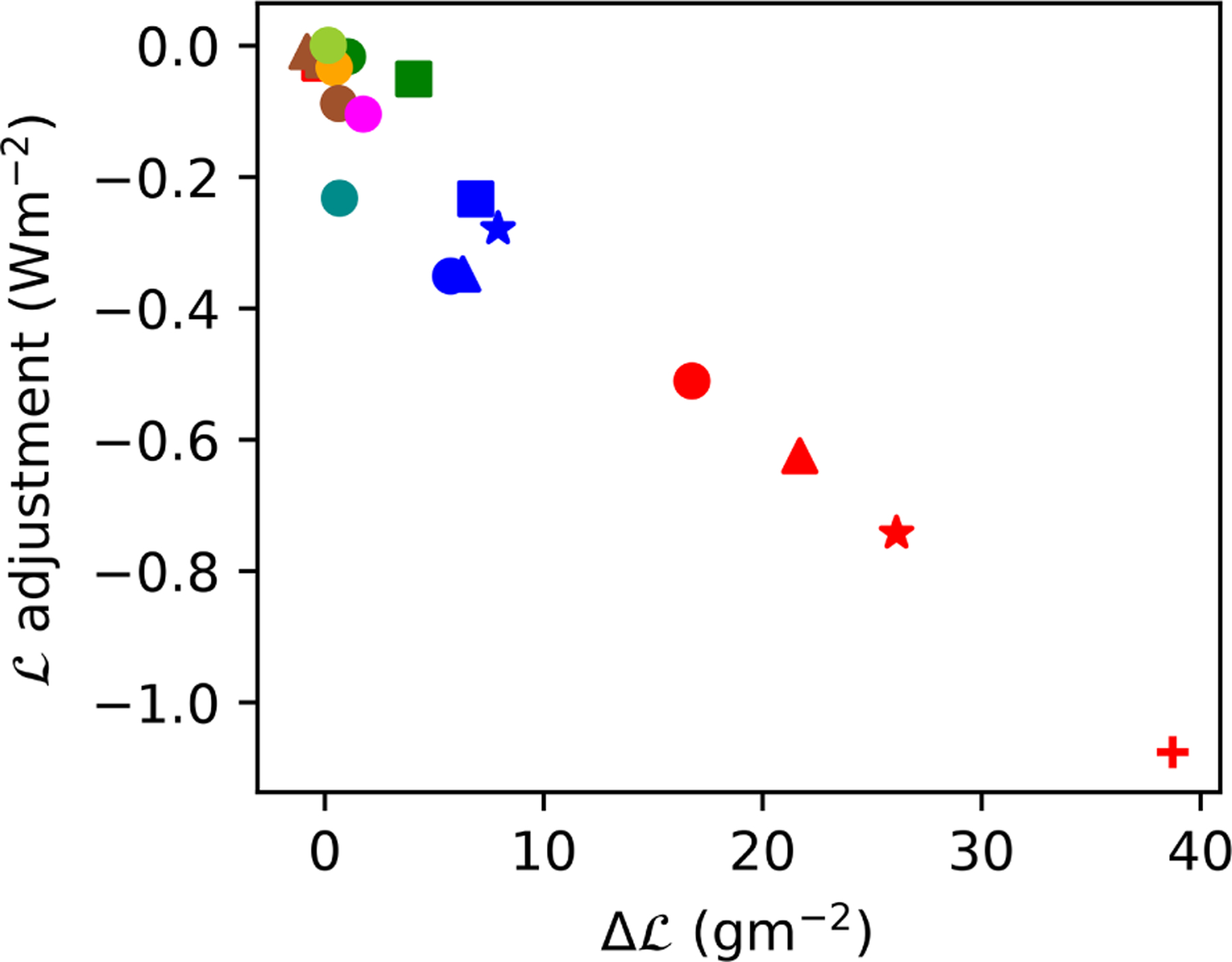
The relationship ΔL (in-cloud) and the L adjustment in each of the models.

Observational estimates of L adjustments are difficult to interpret (Neubauer et al., 2017). Several studies have found a L decrease with increased aerosol or *N*_d_, suggesting a negative adjustment (Chen et al., 2014; Christensen et al., 2017; Sato et al., 2018). However, recent work has suggested that this decrease may overestimate the impact of aerosols on L, supporting a weak L response to aerosol (Malavelle et al., 2017; Gryspeerdt et al., 2019; Toll et al., 2019). In contrast, all the models with a significant RFaci also produce a positive L adjustment, enhancing the ERFaci. As with the *f*_l_ adjustments, the L adjustments are smaller in the CMIP5 models, due to the smaller change in L but similar cloud radiative effects (Fig. 3). The CMIP5 models tend to have less sophisticated aerosol schemes (Table 1), which may explain these weaker adjustments. However, as some models with higher levels of sophistication (e.g. UKESM1-A, MRI-CGCM3) also have weak adjustments, model sophistication is not the only factor influencing the strength of the adjustments.

In almost all of the models, the L and *f*_l_ adjustments have the same sign (Fig. 1b). The different sign of the *f*_l_ and L adjustments in the observation-based studies therefore suggests that inclusion of missing processes controlling L, such as aerosol-dependent entrainment (Ackerman et al., 2004; Xue and Feingold, 2006), may be necessary for models to reproduce the observed relationships (e.g. Salzmann et al., 2010; Guo et al., 2011; Zhou and Penner, 2017; Mülmenstädt and Feingold, 2018).

Although the models typically have stronger L and weaker *f*_l_ enhancements to the RFaci that those from observation-based studies, the models with stronger adjustments have a similar magnitude for the total RFaci enhancement due to adjustments when compared to observations (Fig. 1c). This is an encouraging sign but highlights the potential for models to produce the right answer for the wrong reason.

### 3.4 Ice-cloud ERFaci

**Figure 4.**
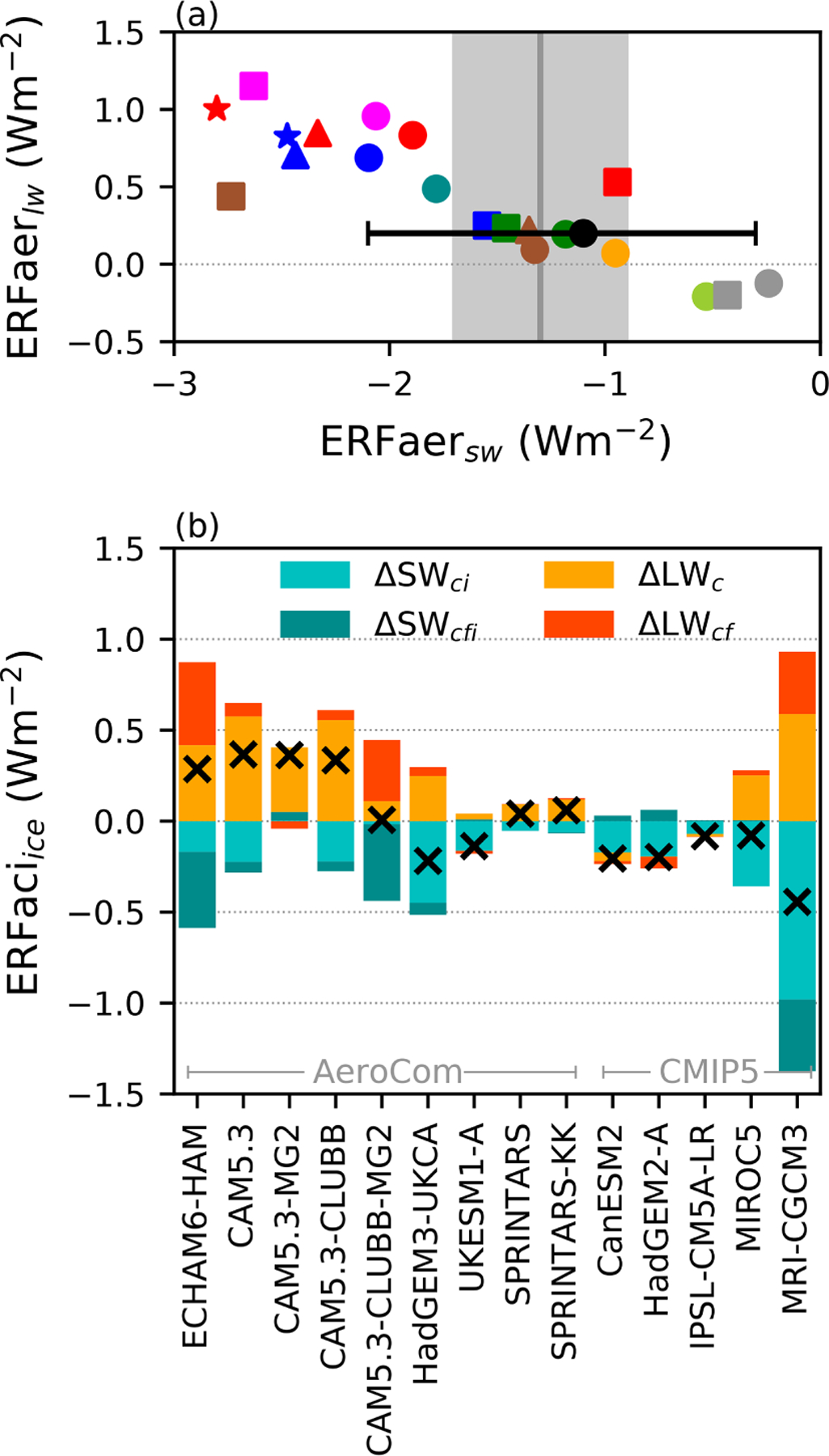
**(a)** The total ERFaer in the longwave as a function of the shortwave ERFaer. The grey range is the estimate from Cherian et al. (2014) and the black circle the expert assessment from Boucher et al. (2013). **(b)** The ERFaci due to changes in ice-cloud properties. Shortwave changes from the cloud albedo (ΔSW_ci_) and ice *f*_c_ (*f*_i_) (ΔSW_cfi_) are shown in blue, including the impact of ice-cloud changes masking lower-level clouds. Longwave changes from changes in intrinsic cloud properties (ΔLW_c_) and cloud fraction (ΔLW_cf_) are in yellow and red, respectively. The cross is the total ERFaci from changes in ice clouds.

As shown in previous modelling studies (Zelinka et al., 2014; Heyn et al., 2017), the model shortwave (SW) and longwave (LW) total aerosol forcings are strongly correlated (Fig. 4a), indicating a strong role of ice clouds, which dominate the longwave aerosol forcing (Table S3). The magnitude of slope of this relationship is smaller than one, such that an increased negative SW forcing is not completely cancelled by a positive LW forcing.

All of the models show an increase in the albedo of ice clouds (Fig. 4b), due to a Twomey-like effect in ice clouds. This is in agreement with current observational studies that suggest an increase in *N*_i_ with an increased aerosol emissions (Gryspeerdt et al., 2018; Mitchell et al., 2018), although there are no current large-scale observational constraints on the forcing from ice clouds. This is offset by a decrease in the outgoing longwave radiation from clouds. These effects occur even in models with no parameterised effect of aerosol directly on convective clouds or ice processes, likely through processes such as droplet freezing.

There is a strong variation in the response of high cloud amount to aerosol between the models. The increase in ice-cloud fraction exhibited by some models produces a negative shortwave forcing (ΔSW_cfi_), but this is closely offset by a positive longwave forcing (ΔLW_cf_), such that the net effect from *f*_i_ changes in high clouds is close to zero. The balance between ΔSW_cfi_ and ΔLW_cf_ varies between the models. The AeroCom models tend to produce a larger longwave effect, resulting in a positive overall forcing (similar to Gettelman et al., 2012), whilst the CMIP5 models generally have an overall forcing close to zero. This may be due to the more detailed representation of clouds and aerosols in the AeroCom models (Table 1). While the AeroCom models are nudged to PD horizontal winds (compared to the free-running CMIP5 models), previous studies show that this does not have a significant impact on the forcing (Zhang et al., 2014) and the negative forcing from UKESM1-A (run with the AeroCom setup) further suggests that model setup does not explain this difference. The variability in the ice-cloud ERFaci is in contrast to the constant adjustment of +0.2 Wm^−2^ used in Boucher et al. (2013), highlighting the current uncertainty in the contribution of ice clouds to the total ERFaer.

## 4 Discussion

The results in this work have shown that when the individual components of the ERFaer are compared, there is an improved agreement between observations and global model estimates. However, there are two important caveats to these results.

The agreement between the observational uncertainty and model diversity, especially for the RFaci (Fig. 1a), is particularly surprising as the RFaci is typically not diagnosed separately from cloud adjustments. Although many models have parameters that can be used to tune the ERFaer, the weak correlation between the ERFaer and the RFaci in the models (*r* = 0.24) further limits the impact of any tuning based on the total aerosol forcing. It should be noted that while the spread in the model RFaci is similar to the spread in the observation-based estimates, many of the models share development pathways (Knutti et al., 2013) and aerosol emissions. Agreement between the models is no guarantee of correctness.

This work also demonstrates that although there is significant variation in the model estimates of the magnitudes of the forcing from liquid cloud adjustments, this variation can be reduced by comparing the adjustments normalised by the RFaci. This accounts for estimates that use a large anthropogenic aerosol fraction (e.g. ECHAM6-HAM2.2-anthsca4), producing a metric that is more closely related to the strength of the liquid cloud adjustments. Uncertainties in observational estimates of the RFaci would introduce uncertainties into the estimate of this enhancement factor, even though uncertainties dependent on the anthropogenic aerosol fraction are significantly reduced by using the enhancement factor. Although there are clear advantages to the RFaci enhancement as a metric for comparing the magnitude of cloud adjustments between models and observation, further work is required to investigate its uncertainty characteristics.

## 5 Conclusions

Previous synthesis studies have found little overlap between distributions of model-based and observation-based estimates of the ERFaer (Boucher et al., 2013). By decomposing the aerosol radiative forcing from GCMs into components, similar to recently developed observational estimates of the ERFaer, this work shows that closer agreement between the model and observational estimates is achieved. In particular, the RFaci in the models investigated is found to be within current observation-based estimates, although there remains significant uncertainty in these observation-based estimates.

The decomposition shows a large variability in the liquid cloud adjustments. The spatial pattern varies from the RFaci pattern, due to the differing physics involved (Fig. 2), but analysing the adjustments as a function of the RFaci mitigates differences from varying aerosol perturbations and droplet activation schemes among the models. Given the large variation in forcing from liquid cloud changes in models, there is a surprising agreement between the model and observational estimates of the RFaci. However, the L and *f*_l_ adjustments show little similarity to current observation-based estimates. This indicates that further work on the observation-based and model estimates is required before they can be relied upon.

There are significant compensations in the longwave radiation from aerosol-induced changes to high and deep clouds, and the sign and magnitude of the overall effect varies significantly between the models, leaving the overall magnitude of the effect uncertain. While early observational studies have indicated a possible negative albedo forcing in the shortwave radiation from changes in the properties of high clouds (e.g. Gryspeerdt et al., 2018; Mitchell et al., 2018), more work is required in this area.

Although the observational and model estimates display a surprising degree of agreement in many cases, a large degree of uncertainty in the ERFaer remains, particularly in the anthropogenic aerosol fraction and in the sensitivity of cloud properties to aerosol. Even where estimates agree, the uncertainties in the model physics and observational estimates mean that this problem is not yet resolved. However, this decomposition provides an encouraging path forward for future studies. This decomposition of the ERFaer is simpler and more computationally efficient to implement than more sophisticated methods (e.g. Mülmenstädt et al., 2019) but closely matches their results. By showing a significant agreement between components of modelled and observational estimates of the aerosol radiative forcing, this study builds confidence in the global model estimates of the aerosol radiative forcing and shows that where model and observation-based studies can be more accurately compared, their similarities become increasingly clear.

## Supplementary Material

Supplementary material

## Figures and Tables

**Table 1.** The ERFaer (global mean differences between the PI and PD TOA radiation) from the AeroCom (top section) and CMIP5 (bottom section) models in watts per square metre (Wm^−2^). CMIP5 physics ensemble members are shown with the “-p” suffix. The third column identifies the nature of the aerosol parameterisation in the model, (0 - direct effect only; 1 - RFaci in liquid clouds, no adjustments; 2 - with liquid cloud adjustments; 3 - parameterised aerosol impacts on ice cloud) following Heyn et al. (2017). Models in italics are sensitivity studies and not included in averages. The icons are used in scatter plots and models of the same family have the same colour. UKESM is not an AeroCom model but has been run in a similar configuration.

Model			Net	Total ΔSW	Total ΔLW
AeroCom indirect effect experiment					
ECHAM6-HAM2.2	●	3	−1.06	−1.89	0.83
– *CND*^1^	■	3	−0.41	−0.94	0.53
– *anthsca1.5*^2^	▲	3	−1.49	−2.33	0.85
– *anthsca2*^2^	★	3	−1.80	−2.80	1.00
– *anthsca4*^2^	+	3	−2.80	−4.24	1.43
CAM5.3	●	3	−1.41	−2.10	0.69
CAM5.3-MG2	■	3	−1.30	−1.55	0.25
CAM5.3-CLUBB	▲	3	−1.73	−2.44	0.70
CAM5.3-CLUBB-MG2	★	3	−1.65	−2.47	0.82
SPRINTARS	●	3	−0.99	−1.18	0.19
SPRINTARS-KK	■	3	−1.23	−1.46	0.23
HadGEM3-UKCA	■	2	−2.30	−2.74	0.44
UKESM1-A	▲	2	−1.13	−1.35	0.22
CMIP5					
CanESM2	●	1	−0.88	−0.95	0.07
HadGEM2-A	●	2	−1.23	−1.33	0.09
IPSL-CM5A-LR	●	1	−0.74	−0.53	−0.21
MIROC5	●	3	−1.30	−1.78	0.49
MRI-CGCM3-p1	●	3	−1.11	−2.06	0.96
MRI-CGCM3-p3^3^	■	3	−1.48	−2.63	1.15
MPI-ESM-LR-p1	●	0	−0.36	−0.24	−0.12
MPI-ESM-LR-p2^4^	■	1	−0.63	−0.43	−0.20
Mean			−1.21	−1.59	0.39

Ensemble key:

1 constant climatological *N*_d_ in autoconversion.

2 scaled anthropogenic emissions.

3 updated cloud scheme.

4 different aerosol forcing data.

**Table 2.** The impact of ice water path thresholds on the RFaci estimate, the forcing from L and *f*_l_ adjustments and the L and *f*_l_ enhancements of the RFaci. The row in bold represents the threshold value used throughout the rest of this work. The bottom rows show the liquid forcing estimates from a simulation with no parameterised cloud adjustment and determined from the standard simulation using the PRP method (Mülmenstädt et al., 2019). Values are in watts per square metre (Wm^−2^) unless otherwise specified.

IWP_min_(gm^−2^)	RFaci	L	*f*_1_	L (%)	*f*_1_ (%)
None	−0.29	−0.37	−0.29	127	153
1	−0.43	−0.50	−0.29	116	67
5	−0.43	−0.51	−0.29	119	67
**8.7** (satellite)	**−0.43**	**−0.51**	**−0.29**	**119**	**67**
10	−0.43	−0.51	−0.29	119	67
25	−0.44	−0.52	−0.29	118	66
100	−0.53	−0.60	−0.29	113	55
CND	−0.42	−0.03	0.07	7	−16
PRP	−0.51	−0.53	−0.31	104	61

## References

[R1] Ackerman AS, Kirkpatrick MP, Stevens DE, and Toon OB: The impact of humidity above stratiform clouds on indirect aerosol climate forcing, Nature, 432, 1014, 10.1038/nature03174, 2004. 15616559

[R2] Ackerman SA, Holz RE, Frey R, Eloranta EW, Maddux BC, and McGill M: Cloud Detection with MODIS, Part II: Validation, J. Atmos. Ocean. Tech, 25, 1073–1086, 10.1175/2007JTECHA1053.1, 2008.

[R3] Albrecht BA: Aerosols, Cloud Microphysics, and Fractional Cloudiness, Science, 245, 1227–1230, 10.1126/science.245.4923.1227, 1989. 17747885

[R4] Andersen H, Cermak J, Fuchs J, Knutti R, and Lohmann U: Understanding the drivers of marine liquid-water cloud occurrence and properties with global observations using neural networks, Atmos. Chem. Phys, 17, 9535–9546, 10.5194/acp-17-9535-2017, 2017.

[R5] Bellouin N, Mann GW, Woodhouse MT, Johnson C, Carslaw KS, and Dalvi M: Impact of the modal aerosol scheme GLOMAP-mode on aerosol forcing in the Hadley Centre Global Environmental Model, Atmos. Chem. Phys, 13, 3027–3044, 10.5194/acp-13-3027-2013, 2013.

[R6] Boucher O, Randall DA, Artaxo P, Bretherton C, Feingold G, Forster PM, Kerminen V-M, Kondo Y, Liao H, Lohmann U, Rasch P, Satheesh SK, Sherwood S, Stevens B, and Zhang XY: Clouds and Aerosols, Cambridge University Press, 10.1017/CBO9781107415324.016, 2013.

[R7] Carslaw KS, Gordon H, Hamilton DS, Johnson JS, Regayre LA, Yoshioka M, and Pringle KJ: Aerosols in the Pre-industrial Atmosphere, Curr. Clim. Change Rep, 3, 1–15, 10.1007/s40641-017-0061-2, 2017. 32226722 PMC7089647

[R8] Chen Y-C, Christensen MW, Stephens GL, and Seinfeld JH: Satellite-based estimate of global aerosol-cloud radiative forcing by marine warm clouds, Nat. Geosci, 7, 643–646, 10.1038/NGEO2214, 2014.

[R9] Cherian R, Quaas J, Salzmann M, and Wild M: Pollution trends over Europe constrain global aerosol forcing as simulated by climate models, Geophys. Res. Lett, 41, 2176–2181, 10.1002/2013GL058715, 2014.

[R10] Christensen MW, Neubauer D, Poulsen CA, Thomas GE, McGarragh GR, Povey AC, Proud SR, and Grainger RG: Unveiling aerosol-cloud interactions - Part 1: Cloud contamination in satellite products enhances the aerosol indirect forcing estimate, Atmos. Chem. Phys, 17, 13151–13164, 10.5194/acp-17-13151-2017, 2017.

[R11] Engström A, Bender FA-M, Charlson RJ, and Wood R: Geographically coherent patterns of albedo enhancement and suppression associated with aerosol sources and sinks, Tellus B, 67, 26442, 10.3402/tellusb.v67.26442, 2015.

[R12] Feingold G: First measurements of the Twomey indirect effect using ground-based remote sensors, Geophys. Res. Lett, 30, 1287, 10.1029/2002GL016633, 2003.

[R13] Fiedler S, Stevens B, and Mauritsen T: On the sensitivity of anthropogenic aerosol forcing to model-internal variability and parameterizing a Twomey effect, J. Adv. Model. Earth Sy, 9, 1325–1341, 10.1002/2017MS000932, 2017.

[R14] Gettelman A: Putting the clouds back in aerosol-cloud interactions, Atmos. Chem. Phys, 15, 12397–12411, 10.5194/acp-15-12397-2015, 2015.

[R15] Gettelman A, Liu X, Barahona D, Lohmann U, and Chen C: Climate impacts of ice nucleation, J. Geophys. Res, 117, D20201, 10.1029/2012JD017950, 2012.

[R16] Ghan S, Wang M, Zhang S, Ferrachat S, Gettelman A, Griesfeller J, Kipling Z, Lohmann U, Morrison H, Neubauer D, Partridge DG, Stier P, Takemura T, Wang H, and Zhang K: Challenges in constraining anthropogenic aerosol effects on cloud radiative forcing using present-day spatiotemporal variability, P. Natl. Acad. Sci. USA, 113, 5804–5811, 10.1073/pnas.1514036113, 2016. PMC4889346 26921324

[R17] Ghan SJ: Technical Note: Estimating aerosol effects on cloud radiative forcing, Atmos. Chem. Phys, 13, 9971–9974, 10.5194/acp-13-9971-2013, 2013.

[R18] Gryspeerdt E, Quaas J, and Bellouin N: Constraining the aerosol influence on cloud fraction, J. Geophys. Res, 121, 3566–3583, 10.1002/2015JD023744, 2016.

[R19] Gryspeerdt E, Quaas J, Ferrachat S, Gettelman A, Ghan S, Lohmann U, Morrison H, Neubauer D, Partridge DG, Stier P, Takemura T, Wang H, Wang M, and Zhang K: Constraining the instantaneous aerosol influence on cloud albedo, P. Natl. Acad. Sci. USA, 114, 4899–4904, 10.1073/pnas.1617765114, 2017. PMC5441736 28446614

[R20] Gryspeerdt E, Sourdeval O, Quaas J, Delanoë J, Krämer M, and Kühne P: Ice crystal number concentration estimates from lidar-radar satellite remote sensing - Part 2: Controls on the ice crystal number concentration, Atmos. Chem. Phys, 18, 14351–14370, 10.5194/acp-18-14351-2018, 2018.

[R21] Gryspeerdt E, Goren T, Sourdeval O, Quaas J, Mülmenstädt J, Dipu S, Unglaub C, Gettelman A, and Christensen M: Constraining the aerosol influence on cloud liquid water path, Atmos. Chem. Phys, 19, 5331–5347, 10.5194/acp-19-5331-2019, 2019.

[R22] Guo H, Golaz J-C, and Donner LJ: Aerosol effects on stratocumulus water paths in a PDF-based parameterization, Geophys. Res. Lett, 38, L17808, 10.1029/2011GL048611, 2011.

[R23] HadGEM3: https://code.metoffice.gov.uk/, last access: 1 May 2019.

[R24] Han Q, Rossow WB, Zeng J, and Welch R: Three Different Behaviors of Liquid Water Path of Water Clouds in Aerosol-Cloud Interactions, J. Atmos. Sci, 59, 726–735, 10.1175/1520-0469(2002)059<0726:TDBOLW>2.0.CO;2, 2002.

[R25] Hasekamp OP, Gryspeerdt E, and Quaas J: Analysis of polarimetric satellite measurements suggests stronger cooling due to aerosol-cloud interactions, Nat. Commun, 10, 1–7, 10.1038/s41467-019-13372-2, 2019. 31776336 PMC6881401

[R26] Heymsfield AJ, Matrosov S, and Baum B: Ice Water Path-Optical Depth Relationships for Cirrus and Deep Stratiform Ice Cloud Layers, J. Appl. Meteorol, 42, 1369–1390, 10.1175/15200450(2003)042<1369:IWPDRF>2.0.CO;2, 2003.

[R27] Heyn I, Block K, Mülmenstädt J, Gryspeerdt E, Kühne P, Salzmann M, and Quaas J: Assessment of simulated aerosol effective radiative forcings in the terrestrial spectrum, Geophys. Res. Lett, 44, 1001–1007, 10.1002/2016GL071975, 2017.

[R28] Kaufman YJ, Koren I, Remer LA, Rosenfeld D, and Rudich Y: The effect of smoke, dust, and pollution aerosol on shallow cloud development over the Atlantic Ocean, P. Natl. Acad. Sci. USA, 102, 11207, 10.1073/pnas.0505191102, 2005. PMC1182178 16076949

[R29] Kinne S: Aerosol radiative effects with MACv2, Atmos. Chem. Phys, 19, 10919–10959, 10.5194/acp-19-10919-2019, 2019.

[R30] Knutti R, Masson D, and Gettelman A: Climate model genealogy: Generation CMIP5 and how we got there, Geophys. Res. Lett, 40, 1194–1199, 10.1002/grl.50256, 2013.

[R31] Lebsock M, Stephens G, and Kummerow C: Multisensor satellite observations of aerosol effects on warm clouds, J. Geophys. Res, 113, D15205, 10.1029/2008JD009876, 2008.

[R32] Malavelle FF, Haywood JM, Jones A, Gettelman A, Clarisse L, Bauduin S, Allan RP, Karset IHH, Kristjánsson JE, Oreopoulos L, Cho N, Lee D, Bellouin N, Boucher O, Grosvenor DP, Carslaw KS, Dhomse S, Mann GW, Schmidt A, Coe H, Hartley ME, Dalvi M, Hill AA, Johnson BT, Johnson CE, Knight JR, O’Connor FM, Partridge DG, Stier P, Myhre G, Platnick S, Stephens GL, Takahashi H, and Thordarson T: Strong constraints on aerosol-cloud interactions from volcanic eruptions, Nature, 546, 485–491, 10.1038/nature22974, 2017. 28640263

[R33] McCoy DT, Bender FA-M, Mohrmann JKC, Hartmann DL, Wood R, and Grosvenor DP: The global aerosol-cloud first indirect effect estimated using MODIS, MERRA, and AeroCom, J. Geophys. Res, 122, 1779–1796, 10.1002/2016JD026141, 2017.

[R34] McCoy DT, Field PR, Schmidt A, Grosvenor DP, Bender FA-M, Shipway BJ, Hill AA, Wilkinson JM, and Elsaesser GS: Aerosol midlatitude cyclone indirect effects in observations and high-resolution simulations, Atmos. Chem. Phys, 18, 5821–5846, 10.5194/acp-18-5821-2018, 2018.

[R35] Mitchell DL, Garnier A, Pelon J, and Erfani E: CALIPSO (IIR-CALIOP) retrievals of cirrus cloud ice-particle concentrations, Atmos. Chem. Phys, 18, 17325–17354, 10.5194/acp-18-17325-2018, 2018. 31662738 PMC6818510

[R36] Mulcahy JP, Jones C, Sellar A, Johnson B, Boutle IA, Jones A, Andrews T, Rumbold ST, Mollard J, Bellouin N, Johnson CE, Williams KD, Grosvenor DP, and McCoy DT: Improved Aerosol Processes and Effective Radiative Forcing in HadGEM3 and UKESM1, J. Adv. Model. Earth Sy, 10, 2786–2805, 10.1029/2018MS001464, 2018.

[R37] Mülmenstädt J and Feingold G: The Radiative Forcing of Aerosol-Cloud Interactions in Liquid Clouds: Wrestling and Embracing Uncertainty, Curr. Clim. Change Rep, 4, 23–40, 10.1007/s40641-018-0089-y, 2018.

[R38] Mülmenstädt J, Gryspeerdt E, Salzmann M, Ma P-L, Dipu S, and Quaas J: Separating radiative forcing by aerosol-cloud interactions and fast cloud adjustments in the ECHAM-HAMMOZ aerosol-climate model using the method of partial radiative perturbations, Atmos. Chem. Phys. Discuss, 10.5194/acp-2018-1304, in review, 2019.

[R39] Neubauer D, Christensen MW, Poulsen CA, and Lohmann U: Unveiling aerosol-cloud interactions - Part 2: Minimising the effects of aerosol swelling and wet scavenging in ECHAM6-HAM2 for comparison to satellite data, Atmos. Chem. Phys, 17, 13165–13185, 10.5194/acp-17-13165-2017, 2017.

[R40] Quaas J, Boucher O, and Lohmann U: Constraining the total aerosol indirect effect in the LMDZ and ECHAM4 GCMs using MODIS satellite data, Atmos. Chem. Phys, 6, 947–955, 10.5194/acp-6-947-2006, 2006.

[R41] Quaas J, Boucher O, Bellouin N, and Kinne S: Satellite-based estimate of the direct and indirect aerosol climate forcing, J. Geophys. Res, 113, 05204, 10.1029/2007JD008962, 2008.

[R42] Quaas J, Ming Y, Menon S, Takemura T, Wang M, Penner JE, Gettelman A, Lohmann U, Bellouin N, Boucher O, Sayer AM, Thomas GE, McComiskey A, Feingold G, Hoose C, Kristjánsson JE, Liu X, Balkanski Y, Donner LJ, Ginoux PA, Stier P, Grandey B, Feichter J, Sednev I, Bauer SE, Koch D, Grainger RG, Kirkevåg A, Iversen T, Seland Ø, Easter R, Ghan SJ, Rasch PJ, Morrison H, Lamarque J-F, Iacono MJ, Kinne S, and Schulz M: Aerosol indirect effects - general circulation model intercomparison and evaluation with satellite data, Atmos. Chem. Phys, 9, 8697–8717, 10.5194/acp-9-8697-2009, 2009.

[R43] Quaas J, Stevens B, Stier P, and Lohmann U: Interpreting the cloud cover - aerosol optical depth relationship found in satellite data using a general circulation model, Atmos. Chem. Phys, 10, 6129–6135, 10.5194/acp-10-6129-2010, 2010.

[R44] Salzmann M, Ming Y, Golaz J-C, Ginoux PA, Morrison H, Gettelman A, Krämer M, and Donner LJ: Two-moment bulk stratiform cloud microphysics in the GFDL AM3 GCM: description, evaluation, and sensitivity tests, Atmos. Chem. Phys, 10, 8037–8064, 10.5194/acp-10-8037-2010, 2010.

[R45] Sato Y, Goto D, Michibata T, Suzuki K, Takemura T, Tomita H, and Nakajima T: Aerosol effects on cloud water amounts were successfully simulated by a global cloud-system resolving model, Nat. Commun, 9, 1–7, 10.1038/s41467-018-03379-6, 2018. 29515125 PMC5841301

[R46] Schutgens N, Tsyro S, Gryspeerdt E, Goto D, Weigum N, Schulz M, and Stier P: On the spatio-temporal representativeness of observations, Atmos. Chem. Phys, 17, 9761–9780, 10.5194/acp-17-9761-2017, 2017.

[R47] Seifert A, Heus T, Pincus R, and Stevens B: Large-eddy simulation of the transient and near-equilibrium behavior of precipitating shallow convection, J. Adv. Model. Earth Sy, 7, 1918–1937, 10.1002/2015MS000489, 2015.

[R48] Sellar AA, Jones CG, Mulcahy J, Tang Y, Yool A, Wiltshire A, O’Connor FM, Stringer M, Hill R, Palmieri J, Woodward S, Mora L, Kuhlbrodt T, Rumbold S, Kelley DI, Ellis R, Johnson CE, Walton J, Abraham NL, Andrews MB, Andrews T, Archibald AT, Berthou S, Burke E, Blockley E, Carslaw K, Dalvi M, Edwards J, Folberth GA, Gedney N, Griffiths PT, Harper AB, Hendry MA, Hewitt AJ, Johnson B, Jones A, Jones CD, Keeble J, Liddicoat S, Morgenstern O, Parker RJ, Predoi V, Robertson E, Siahaan A, Smith RS, Swaminathan R, Woodhouse MT, Zeng G, and Zerroukat M: UKESM1: Description and evaluation of the UK Earth System Model, J. Adv. Model. Earth Sy, 11, 10.1029/2019MS001739, 2019.

[R49] Stevens B: Rethinking the Lower Bound on Aerosol Radiative Forcing, J. Clim, 28, 4794–4819, 10.1175/JCLI-D-14-00656.1, 2015.

[R50] Stier P: Limitations of passive remote sensing to constrain global cloud condensation nuclei, Atmos. Chem. Phys, 16, 6595–6607, 10.5194/acp-16-6595-2016, 2016.

[R51] Toll V, Christensen M, Quaas J, and Bellouin N: Weak average liquid-cloud-water response to anthropogenic aerosols, Nature, 572, 51–55, 10.1038/s41586-019-1423-9, 2019. 31367029

[R52] Twomey S: Pollution and the planetary albedo, Atmos. Environ, 8, 1251–1256, 10.1016/0004-6981(74)90004-3, 1974.

[R53] Xue H and Feingold G: Large-Eddy Simulations of Trade Wind Cumuli: Investigation of Aerosol Indirect Effects, J. Atmos. Sci, 63, 1605–1622, 10.1175/JAS3706.1, 2006.

[R54] Zelinka MD, Andrews T, Forster PM, and Taylor KE: Quantifying components of aerosol-cloud-radiation interactions in climate models, J. Geophys. Res, 119, 7599–7615, 10.1002/2014JD021710, 2014.

[R55] Zhang K, Wan H, Liu X, Ghan SJ, Kooperman GJ, Ma P-L, Rasch PJ, Neubauer D, and Lohmann U: Technical Note: On the use of nudging for aerosol-climate model intercomparison studies, Atmos. Chem. Phys, 14, 8631–8645, 10.5194/acp-14-8631-2014, 2014.

[R56] Zhang S, Wang M, Ghan SJ, Ding A, Wang H, Zhang K, Neubauer D, Lohmann U, Ferrachat S, Takeamura T, Gettelman A, Morrison H, Lee Y, Shindell DT, Partridge DG, Stier P, Kipling Z, and Fu C: On the characteristics of aerosol indirect effect based on dynamic regimes in global climate models, Atmos. Chem. Phys, 16, 2765–2783, 10.5194/acp-16-2765-2016, 2016.

[R57] Zhou C and Penner JE: Why do general circulation models overestimate the aerosol cloud lifetime effect? A case study comparing CAM5 and a CRM, Atmos. Chem. Phys, 17, 21–29, 10.5194/acp-17-21-2017, 2017.

