# Supplementary material for: Surprising similarities in model and observational aerosol radiative forcing estimates
Source: Atmos Chem Phys. Author manuscript; Available in PMC 2020 Nov 16. (PMC7668122; doi:10.5194/acp-20-613-2020)
Supplement: Supplementary material [file NIHMS1643833-supplement-Supplementary_material.pdf]

Supplement of Atmos. Chem. Phys., 20, 613–623, 2020
https://doi.org/10.5194/acp-20-613-2020-supplement
© Author(s) 2020. This work is distributed under
the Creative Commons Attribution 4.0 License.

Supplement of

Surprising similarities in model and observational aerosol radiative forcing estimates

Edward Gryspeerdt et al.

Correspondence to: Edward Gryspeerdt (e.gryspeerdt@imperial.ac.uk)

The copyright of individual parts of the supplement might differ from the CC BY 4.0 License.

Supplementary information

S1 Required output

Two simulations are required: one present day and one pre-industrial. If the simulations are nudged to present day meteorology, five years for each is sufficient. A list of required variables is given in Tab. S1.

S2 The RFari

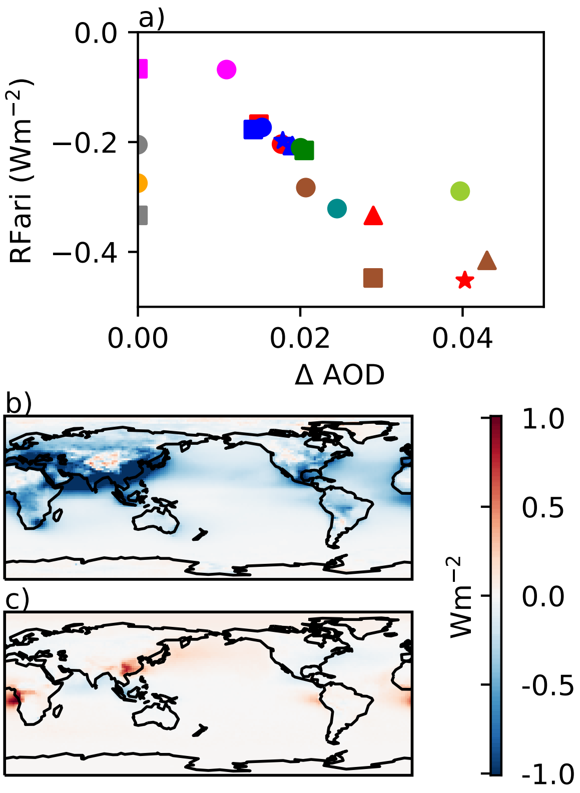

Figure S1. a) The change in AOD against the RFari (clear-sky), model icons are given in Table 1. b) The ensemble mean shortwave RFari (clear-sky). c) The mean shortwave RFari (cloudy-sky) for the AeroCom models.

The RFari is closely related to the change in the AOD (Fig. S1a), with an overall correlation between the RFari and the mean change in AOD of -0.76. The harmonized emissions from the AeroCom models (red, blue and green icons) produce a tight grouping of RFari estimates around $-0.2\,\mathrm{W\,m^{-2}}$, demonstrating the important role of the anthropogenic aerosol fraction. Although the role of surface albedo changes is generally small, it cannot always be neglected, almost doubling the RFari in the SPRINTARS model (SI Tab. S2). The impact of aerosol in overcast locations (RFari_c) is much smaller, averaging $+0.06\,\mathrm{W\,m^{-2}}$ in the AeroCom models. The CMIP5 models did not produce suitable output to calculate the RFari_c, but comparison to the

absorbing component of the aerosol–cloud interaction in Zelinka et al. (2014) suggests that it is likely to also be small. The spatial patterns of the RFari and the RFari_c are very different. The RFari (Fig. S1b) is strongest in regions of large aerosol perturbations, while the RFari_c (Fig. S1c) is stronger in regions with large amounts of low-level liquid cloud. The overall mean estimate of $-0.2\,\text{W}\,\text{m}^{-2}$ is smaller than the $-0.27\,\text{W}\,\text{m}^{-2}$ from Quaas et al. (2009), but some of this difference is due to the postive forcing from aerosol above clouds.

S3 Comparisons to the PRP method

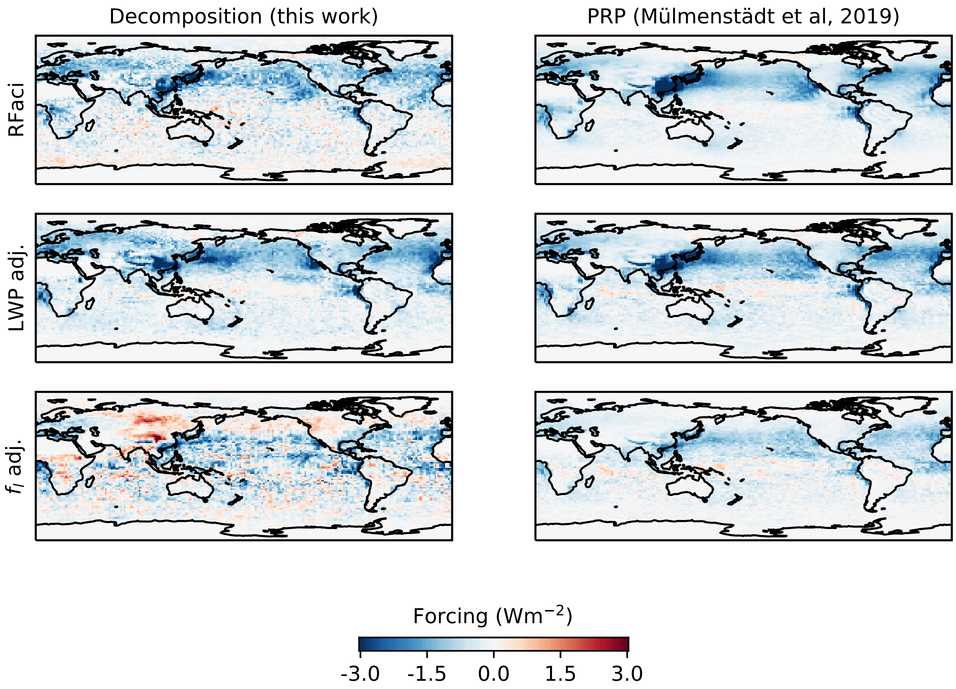

Figure S2. Comparison of the spatial patterns of the forcing from the three components of the ERFaci for liquid clouds: RFaci (top row), the LWP adjustment (middle row) and the liquid f_c adjustment (bottom row). The decomposition in this work is shown on the left and the PRP (partial radiative perturbation method) from Mülmenstädt et al. (2019) is shown on the right.

There is a close match between the spatial patterns of the liquid cloud forcing components between the method proposed in this work and the partial radiative perturbation (PRP) method from Mülmenstädt et al. (2019). The positive forcings for the liquid cloud adjustments in the decomposition in this work are the remainder of the method for isolating changes in f_l from overlying f_i changes.

CMOR name	Long name/*Notes*
rsut	TOA outgoing shortwave (all-sky)
	All-sky albedo is an alternative
rsutcs	TOA outgoing shortwave (clear-sky)
	Clear-sky albedo is an alternative
clt	Total cloud fraction
clwvi	Liquid water path
clivi	Ice water path
icc	Ice cloud fraction
	Satellite simulated fraction
clhcalipso	CALIPSO simulator high cloud fraction
	Alternative to icc
clmcalipso	CALIPSO simulator mid-level cloud fraction
	Alternative to icc
lcc	Liquid cloud fraction
	Satellite simulated fraction
cllcalipso	CALIPSO simulator low cloud fraction
	Alternative to lcc
rlut	TOA Outgoing longwave radiation (all-sky)
	Longwave/ice decomposition only
rlutcs	TOA Outgoing longwave radiation (clear-sky)
	Longwave/ice decomposition only
rsdt	TOA incoming shortwave
	Can be calculated offline
	Not required if albedos used
rsutnoa	rsut but with aerosol not active in radiation
rsutcsnoa	rsutcs but with aerosol not active in radiation
	Required for above-cloud aerosol
rsdscs	Downwelling shortwave at surface
	Required for surface if rsutcsnoa unavailable
rsuscs	Upwelling shortwave at surface
	Required for surface if rsutcsnoa unavailable
od550aer	Aerosol optical depth at 550nm
	Analysis only, not required for decomposition

Table S1. Required output variables for the decomposition

Model	ΔSW	ΔSurf.	$SWari_{cs}$	$SWari_{cld}$	ΔSW_{cl}	ΔSW_{ci}	ΔSW_{cfl}	ΔSW_{cfi}	Res
ECHAM6-HAM2.2	−1.89	−0.01	−0.20	0.03	−0.94	−0.17	−0.17	−0.42	−0.01
-CND	−0.94	0.00	−0.17	-	−0.45	−0.10	0.18	−0.37	−0.03
-anthsca1.5	−2.33	0.00	−0.33	-	−1.09	−0.20	−0.19	−0.49	−0.03
-anthsca2	−2.80	0.00	−0.45	-	−1.28	−0.24	−0.23	−0.58	−0.02
-anthsca4	−4.24	0.00	−0.89	-	−1.83	−0.35	−0.36	−0.82	0.01
CAM5.3	−2.10	0.01	−0.17	0.12	−1.13	−0.22	−0.68	−0.06	0.04
CAM5.3-MG2	−1.55	−0.01	−0.18	0.08	−1.29	0.00	−0.11	0.05	−0.08
CAM5.3-CLUBB	−2.44	0.00	−0.21	0.11	−1.22	−0.22	−0.97	−0.05	0.12
CAM5.3-CLUBB-MG2	−2.47	0.00	−0.20	0.08	−1.44	−0.02	−0.64	−0.42	0.16
SPRINTARS	−1.18	−0.18	−0.21	−0.03	−0.68	−0.05	−0.06	−0.00	0.03
SPRINTARS-KK	−1.46	−0.19	−0.22	−0.03	−0.79	−0.06	−0.15	−0.00	−0.01
HadGEM3-UKCA	−2.74	-	−0.45	-	−1.29	−0.45	−0.18	−0.07	−0.30
UKESM1-A	−1.35	0.00	−0.42	−0.06	−0.56	−0.16	−0.22	0.01	0.05
CanESM2	−0.95	−0.01	−0.27	-	−0.47	−0.17	−0.06	0.03	0.01
HadGEM2-A	−1.33	0.02	−0.28	-	−0.76	−0.19	−0.27	0.06	0.10
IPSL-CM5A-LR	−0.53	−0.03	−0.29	-	−0.07	−0.07	−0.07	0.00	−0.00
MIROC5	−1.78	−0.05	−0.32	-	−1.16	−0.36	−0.23	0.00	0.33
MRI-CGCM3-p1	−2.06	0.03	−0.07	-	−0.82	−0.98	−0.05	−0.39	0.22
MRI-CGCM3-p3	−2.63	0.03	−0.07	-	-	-	-	-	-
MPI-ESM-LR-p1	−0.24	−0.01	−0.20	-	-	-	-	-	-
MPI-ESM-LR-p3	−0.43	−0.05	−0.33	-	-	-	-	-	-
Mean	−1.78	−0.04	−0.26	0.01	−0.96	−0.22	−0.25	−0.20	0.03
S.D.	0.93	0.11	0.18	0.04	0.41	0.22	0.26	0.26	0.13

Table S2. The SW decomposition. The column "Res" is the residual from the decomposition into changes in cloud albedo "$\Delta\alpha_c$" and cloud fraction Δf_c. Values are given in W m^{-2}, with a negative indicating a negative radiative forcing (a cooling effect). Dashes indicate insufficient output for the decomposition, these components are assumed zero in calculating the residuals. CMIP5 values assume no impact of aerosol above cloud.

Model	ΔLW	LWari$_{cs}$	ΔLW$_{cl}$	ΔLW$_{ci}$	ΔLW$_{cfl}$	ΔLW$_{cfi}$	Res
ECHAM6-HAM2.2	0.83	0.01	−0.04	0.42	0.03	0.46	−0.05
-CND	0.53	−0.03	−0.08	0.31	−0.07	0.42	−0.02
-anthsca1.5	0.85	0.01	−0.10	0.41	0.04	0.56	−0.07
-anthsca2	1.00	0.02	−0.12	0.48	0.05	0.67	−0.10
-anthsca4	1.43	0.06	−0.22	0.65	0.08	0.98	−0.11
CAM5.3	0.69	0.09	−0.07	0.58	0.19	0.07	−0.17
CAM5.3-MG2	0.25	0.05	0.01	0.35	0.10	−0.04	−0.22
CAM5.3-CLUBB	0.70	0.10	−0.08	0.56	0.29	0.05	−0.21
CAM5.3-CLUBB-MG2	0.82	0.12	0.03	0.11	0.21	0.34	0.01
SPRINTARS	0.19	0.07	0.01	0.09	0.02	0.00	−0.00
SPRINTARS-KK	0.23	0.08	0.00	0.12	0.05	0.01	−0.02
HadGEM3-UKCA	0.44	0.11	0.09	0.25	0.06	0.05	−0.12
UKESM1-A	0.22	0.14	0.09	0.03	0.04	−0.02	−0.07
CanESM2	0.07	0.06	0.04	−0.05	0.02	−0.02	0.02
HadGEM2-A	0.09	0.08	0.06	−0.00	0.04	−0.06	−0.02
IPSL-CM5A-LR	−0.21	0.02	−0.01	−0.02	0.02	−0.00	−0.22
MIROC5	0.49	0.12	0.06	0.25	0.06	0.03	−0.03
MRI-CGCM3	0.96	0.00	0.01	0.59	0.01	0.34	0.00
MRI-CGCM3	1.15	0.00	-	-	-	-	-
MPI-ESM-LR	−0.12	−0.01	-	-	-	-	-
MPI-ESM-LR	−0.20	−0.02	-	-	-	-	-
Mean	0.51	0.05	−0.02	0.30	0.07	0.23	−0.07
S.D.	0.45	0.05	0.08	0.22	0.08	0.30	0.08

Table S3. As Tab. S2, but the LW decomposition. The final column shows the residual from the decomposition. Values are in $\mathrm{W\,m^{-2}}$.

Model	N_d	$\mathscr{L}$	f_c	f_c(corr)	$\mathscr{L}$ (%)	f_c(%)	f_c(corr, %)
ECHAM6-HAM2.2	-0.43	-0.51	-0.17	-0.29	118	38	66
-CND	-0.42	-0.03	0.18	0.07	6	-43	-17
-anthsca1.5	-0.46	-0.62	-0.19	-0.33	135	41	72
-anthsca2	-0.54	-0.74	-0.23	-0.39	138	42	73
-anthsca4	-0.76	-1.08	-0.36	-0.60	142	47	79
CAM5.3	-0.78	-0.35	-0.68	-0.70	45	87	89
CAM5.3-MG2	-1.06	-0.23	-0.11	-0.07	22	11	7
CAM5.3-CLUBB	-0.87	-0.35	-0.97	-0.99	40	111	113
CAM5.3-CLUBB-MG2	-1.16	-0.28	-0.64	-0.81	24	55	69
SPRINTARS	-0.66	-0.02	-0.06	-0.06	3	9	9
SPRINTARS-KK	-0.74	-0.05	-0.15	-0.15	7	20	20
HadGEM3-UKCA	-1.26	-0.02	-0.18	-0.19	2	15	15
UKESM1-A	-0.55	-0.01	-0.22	-0.20	2	39	36
CanESM2	-0.44	-0.03	-0.06	-0.05	8	14	12
HadGEM2-A	-0.68	-0.09	-0.27	-0.25	13	40	36
IPSL-CM5A-LR	-0.07	0.00	-0.07	-0.06	-1	97	78
MIROC5	-0.93	-0.23	-0.23	-0.26	25	25	28
MRI-CGCM3	-0.71	-0.10	-0.05	-0.14	15	8	19

Table S4. The decomposition of the forcing from changes in liquid cloud albedo (SW_l) into forcings from $\mathscr{L}$ and N_d (RFaci) changes. The forcing from liquid cloud fraction changes is shown, along with a forcing assuming no ice cloud fraction changes (similar to observation-based studies). Values are in $W\,m^{-2}$.
